# High-Resolution Genome-Wide Occupancy in *Candida* spp. Using ChEC-seq

**DOI:** 10.1128/mSphere.00646-20

**Published:** 2020-10-14

**Authors:** Faiza Tebbji, Inès Khemiri, Adnane Sellam

**Affiliations:** a Montreal Heart Institute, Université de Montréal, Montréal, Quebec, Canada; b Department of Microbiology, Infectious Diseases and Immunology, Faculty of Medicine, Université de Montréal, Montréal, Quebec, Canada; University of Georgia

**Keywords:** *Candida albicans*, *Candida* spp., ChEC-seq, genome-wide occupancy, transcriptional regulatory network

## Abstract

To persist in their dynamic human host environments, fungal pathogens must sense and adapt by modulating their gene expression to fulfill their cellular needs. Understanding transcriptional regulation on a global scale would uncover cellular processes linked to persistence and virulence mechanisms that could be targeted for antifungal therapeutics. Infections associated with the yeast Candida albicans, a highly prevalent fungal pathogen, and the multiresistant related species Candida auris are becoming a serious public health threat. To define the set of a gene regulated by a transcriptional regulator in C. albicans, chromatin immunoprecipitation (ChIP)-based techniques, including ChIP with microarray technology (ChIP-chip) or ChIP-DNA sequencing (ChIP-seq), have been widely used. Here, we describe a new set of PCR-based micrococcal nuclease (MNase)-tagging plasmids for C. albicans and other *Candida* spp. to determine the genome-wide location of any transcriptional regulator of interest using chromatin endogenous cleavage (ChEC) coupled to high-throughput sequencing (ChEC-seq). The ChEC procedure does not require protein-DNA cross-linking or sonication, thus avoiding artifacts related to epitope masking or the hyper-ChIPable euchromatic phenomenon. In a proof-of-concept application of ChEC-seq, we provided a high-resolution binding map of the SWI/SNF chromatin remodeling complex, a master regulator of fungal fitness in C. albicans, in addition to the transcription factor Nsi1 that is an ortholog of the DNA-binding protein Reb1 for which genome-wide occupancy was previously established in Saccharomyces cerevisiae. The ChEC-seq procedure described here will allow a high-resolution genomic location definition which will enable a better understanding of transcriptional regulatory circuits that govern fungal fitness and drug resistance in these medically important fungi.

**IMPORTANCE** Systemic fungal infections caused by Candida albicans and the “superbug” Candida auris are becoming a serious public health threat. The ability of these yeasts to cause disease is linked to their faculty to modulate the expression of genes that mediate their escape from the immune surveillance and their persistence in the different unfavorable niches within the host. Comprehensive knowledge on gene expression control of fungal fitness is consequently an interesting framework for the identification of essential infection processes that could be hindered by chemicals as potential therapeutics. Here, we expanded the use of ChEC-seq, a technique that was initially developed in the yeast model Saccharomyces cerevisiae to identify genes that are modulated by a transcriptional regulator, in pathogenic yeasts from the genus *Candida*. This robust technique will allow a better characterization of key gene expression regulators and their contribution to virulence and antifungal resistance in these pathogenic yeasts.

## Abstract (precis)

Systemic fungal infections caused by Candida albicans and the “superbug” Candida auris are becoming a serious public health threat. The ability of these yeasts to cause disease is linked to their faculty to modulate the expression of genes that mediate their escape from the immune surveillance and their persistence in the different unfavorable niches within the host. Comprehensive knowledge on gene expression control of fungal fitness is consequently an interesting framework for the identification of essential infection processes that could be hindered by chemicals as potential therapeutics. Here, we expanded the use of ChEC-seq, a technique that was initially developed in the yeast model Saccharomyces cerevisiae to identify genes that are modulated by a transcriptional regulator, in pathogenic yeasts from the genus *Candida*. This robust technique will allow a better characterization of key gene expression regulators and their contribution to virulence and antifungal resistance in these pathogenic yeasts.

## INTRODUCTION

*Candida* species, in particular Candida albicans, are major components of the disease burden caused by fungi and are frequent causes of life-threatening invasive infections especially in immunocompromised patients. The emergent Candida auris was the first fungal pathogen considered an urgent public health threat due to its multidrug resistance, high transmissibility among patients in health care facilities, and elevated crude mortality (1). Other *Candida* species such as C. parapsilosis, C. tropicalis, C. guilliermondii, and the azole-resistant yeasts C. glabrata and C. krusei are also frequent causes of candidiasis and vulvovaginal infections (2–4). Current anti-*Candida* therapeutics suffer from diverse limitations, including toxicity, resistance, and interactions with other commonly prescribed drugs. This has led to increasing interest in studying mechanisms underlying resistance and virulence of *Candida* species with the ultimate goal to identify potential drug targets for novel antifungal therapeutic intervention. However, the diploid nature and the absence of a complete sexual cycle in most *Candida* species limit the use of classical genetic approaches to dissect mechanisms controlling fungal fitness and antifungal resistance. Alternatively, applying genome-wide transcriptional methods such as those determining gene expression changes (DNA microarrays and transcriptome sequencing [RNA-seq]) or genomic occupancy (chromatin immunoprecipitation with microarray technology [ChIP-chip] and chromatin immunoprecipitation-DNA sequencing [ChIP-seq]) in *Candida* species had significantly contributed to uncovering different facets of fungal biology that are critical for both opportunistic and commensal lifestyles, in addition to antifungal tolerance and resistance (5–17). These approaches had also helped to uncover a surprising extent of evolutionary plasticity of transcriptional regulatory circuits in these species compared to the model yeast Saccharomyces cerevisiae (18, 19).

While ChIP-chip and ChIP-seq have been traditionally used to unbiasedly map the binding of a transcriptional regulator (TR), this tool has some limitations that are attributed mainly to TR-DNA cross-linking and DNA shearing by sonication (20). Formaldehyde is commonly used for protein-DNA cross-linking; however, this chemical preferentially generates protein-protein cross-links which can cause epitope masking and consequently alters the efficiency of the immunoprecipitation procedure and leads to increased signal background noise. Furthermore, DNA fragmentation by sonication can disrupt weak or transient TR-DNA or TR-histone interactions and generate DNA fragments with heterogenous sizes and thus impede the refinement of binding site identification (21).

To circumvent these limitations, cross-linking- and sonication-free alternative methods have been developed recently (20, 22–25). In one such method termed chromatin endogenous cleavage (ChEC) (26), the TR of interest is fused to the micrococcal nuclease (MNase) in order to fragment unprotected neighboring chromatin upon MNase activation by calcium (26). ChEC coupled to high-throughput sequencing (ChEC-seq) was efficiently used to map the binding of the general transcription factors Reb1, Abf1, and Rap1 in the budding yeast and has provided a high-resolution occupancy with more binding events than ChIP-based tools (20). Additionally, temporal analysis of ChEC-seq data uncovered that TR can have two distinct binding behaviors: a fast binding uncovered by rapid MNase cleavage at a locus with a robust bona fide TR-binding motif and a second slow cleavage with low-scoring motifs that are preferentially sampled by a given TR. ChEC-seq has been successfully used to define genomic occupancy of the chromatin remodeler RSC complex (Rsc8 subunit) as the ChIP procedure was less efficient (27, 28). Several recent investigations took advantage of ChEC-seq to study the role of different core components of the general transcriptional machinery such as mediators, SAGA (Spt-Ada-Gcn5-acetyltransferase), histone acetyltransferases, and chromatin “pushers,” on global gene expression control and promoter nucleosome architecture in eukaryotes (29–32).

In this work, we describe a new set of PCR-based MNase-tagging plasmids for C. albicans and other *Candida* species to determine genome-wide location of any TR of interest by ChEC-seq. In a proof-of-concept application of ChEC-seq in C. albicans, we have selected Nsi1 that is an ortholog of the DNA-binding protein Reb1 for which genome-wide occupancy was previously established by ChEC-seq in S. cerevisiae (20). As our previous effort on mapping occupancy of the C. albicans chromatin remodeling complex SWI/SNF by ChIP-tiling arrays had led to substantial background noise (7), we have used the ChEC-seq assay to obtain a high-resolution binding map of this master regulator of fungal fitness (6). The ChEC-seq procedure described here will allow a high-resolution genomic location definition which will enable a better understanding of transcriptional regulatory circuits that govern fungal fitness and drug resistance in these medically important fungi.

## RESULTS AND DISCUSSION

### Plasmid toolbox for MNase tagging in C. albicans and non-*albicans Candida* species.

**FIG 1:**
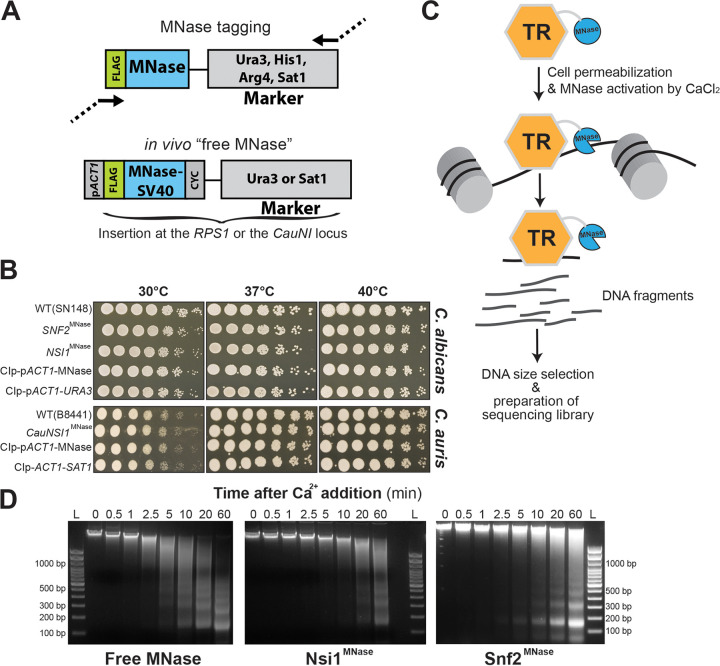
ChEC-seq method in C. albicans and *Candida* spp. (A) Plasmid constructs for *in vivo* TR-MNase tagging and the construction of the “free MNase” control strains in C. albicans and C. auris. Structure of the MNase-tagging cassette consisting of a 3xFLAG epitope fused to the C. albicans codon-optimized MNase. The FLAG-MNase construct for the “free MNase” strain was constitutively expressed using the C. albicans *ACT1* promoter (p*ACT1*). CYC, *CYC1* terminator; SV40, nuclear localization signal. (B) Phenotypic characterization of strains bearing the MNase-tagged Nsi1 and Snf2 TRs and the “free MNase” control constructs in C. albicans. WT, Snf2^MNase^, Nsi1^MNase^, the free MNase, and the control (empty vector) strains were serially diluted, spotted on YPD, and incubated for 1 day at different temperatures. Growth patterns at different temperatures of the C. auris WT, CauNsi1^MNase^, the free MNase and the control strains are also shown. (C) Schematic representation of the experimental setup of the ChEC-seq methodology. *Candida* species cells where a TR of interest is fused to MNase are permeabilized with digitonin prior to MNase activation with calcium. This will lead to the fragmentation of unprotected neighboring chromatin. The resulting fragmented DNA is purified and subjected to size selection prior to high-throughput sequencing. (D) Evaluation of genomic DNA fragmentation by agarose gel electrophoresis at 0, 0.5, 1, 2.5, 5, 10, 20, and 60 min of calcium exposure in the Snf2^MNase^, Nsi1^MNase^, and the free MNase strains in C. albicans. L, 100-bp DNA ladder.

We have previously constructed a series of pFA plasmids for C-terminal hemagglutinin (HA), tandem affinity purification (TAP), and MYC tagging in C. albicans with the *URA3*, *HIS1*, and *ARG4* autotrophy markers (33). Here, we have used these plasmids as a starting point to build new pFA plasmids that allow C-terminal tagging of any protein of interest at its native chromosomal location with the MNase. We synthesized a DNA construct encoding a 3xFLAG epitope and MNase that have been codon optimized for C. albicans. This construct was used to replace the DNA sequence of the TAP tag in pFA-TAP-CaURA3, pFA-TAP-CaHIS1, and pFA-TAP-CaARG4 to generate pFA-MNase-CaURA3, pFA-MNase-CaHIS1, and pFA-MNase-CaARG4, respectively. These plasmids allow the use of a single 120-bp primer pair (20 bp of vector sequences and 100 bp from the gene to be tagged) for PCR-based tagging of endogenous loci in C. albicans (Fig. 1A). These primers are compatible with the pFA-TAP/HA/MYC (33) and the pFA-XFP tagging systems (34, 35). We have also constructed the pFA-MNase-SAT1 plasmid with the dominant selectable marker *SAT1* that confers resistance to the antibiotic nourseothricin for MNase tagging in clinical strains of C. albicans and non-*albicans Candida* species such as the superbug C. auris.

### ChEC-seq experimental procedure.

ChEC-seq was initially used in S. cerevisiae to map the genomic occupancy of canonical general regulatory factors such as the RNA polymerase I enhancer-binding protein Reb1 (ScReb1) (20). Here, we have selected Nsi1 (C6_03550C_A), which is the ScReb1 ortholog in C. albicans, to perform ChEC-seq. Additionally, we were also interested in the catalytic subunit of the SWI/SNF complex, Snf2, to explore the potential of ChEC-seq in mapping genomic occupancy of chromatin remodeling complexes. We have previously mapped the genomic location of C. albicans Snf6, which is a fungus-specific SWI/SNF subunit, using ChIP coupled to high-density tiling arrays (7). Thus, the SWI/SNF genome-wide binding data generated by ChEC-seq can be compared to the benchmark data sets generated by ChIP-chip. We also generated an MNase control strain (“free MNase”) with a 3xFLAG-tagged MNase module fused to a simian virus 40 (SV40) nuclear localization signal under the control of *ACT1* and integrated at the *RPS1* locus. Constitutive expression of MNase (free MNase) or MNase tagging of Nsi1 or Snf2 had no perceptible effect on the growth of C. albicans at different temperatures and stress conditions (Fig. 1B; see also Fig. S1 in the supplemental material).

10.1128/mSphere.00646-20.1 FIG S1 Phenotypic characterization of strains bearing the MNase-tagged transcriptional regulators, and the “free MNase” control constructs in C. albicans and C. auris. Strains were grown with different stresses as described in Materials and Methods. The relative growth was determined as the OD ratio of the YPD control to the treated cultures and is expressed as a percentage. The results are the means of the results from at least three biological replicates. Statistical significance was tested using Student’s *t* test. NS, not significant. Download FIG S1, TIF file, 0.5 MB. Copyright © 2020 Tebbji et al. 2020 Tebbji et al. This content is distributed under the terms of the Creative Commons Attribution 4.0 International license.

The Nsi1 ortholog of C. auris was also MNase tagged using PCR cassettes generated from the pFA-MNase-SAT1 plasmid. For the MNase control strain, a 3xFLAG-tagged MNase module was inserted into the neutral intergenic locus *CauNI* where integration has no effect on the *in vitro* fitness of C. auris (Fig. 1B). As for C. albicans, MNase tagging of CauNsi1 does not affect the growth of C. auris (Fig. 1B and Fig. S1).

The S. cerevisiae ChEC procedure described by Zentner et al. (20) was followed with some modifications (Fig. 1C). C. albicans cells were permeabilized with digitonin for 10 min prior to MNase activation with 5 mM CaCl_2_. We assumed that treating permeabilized cells with calcium would engender both specific and nonspecific cleavages. We therefore made a size selection of the ChEC DNA before preparing the sequencing library to enrich small fragments less than 400 bp. Prior to size selection and for each transcriptional regulator and the free MNase strain, we analyzed the kinetics of DNA digestion by agarose gel electrophoresis. Analysis of minute-scale time points revealed notable smearing of genomic DNA of all TF-MNase fusions by the 5-min time point. This pattern increased over time until 60 min. In contrast, digestion in the free MNase strain yield smearing as early as 30 s (Fig. 1D). The 5-, 20-, and 60-min digestion times were selected for both Snf2 and Nsi1 ChEC-seq experiments. Size selection was performed using the Pippin Prep size selection system with 2% agarose gel cassette. The goal of this stage is to remove multikilobase fragments of genomic DNA and enrich small fragments. The 2% agarose gel cassette allows enrichment of DNA fragments below 100 to 400 bp. Alternatively, size selection could be performed using the paramagnetic beads and buffer exchange steps such as the AMPure XP cleanup kit (Beckman coulter) (20, 36). For C. auris, similar cell permeabilization and MNase activation procedures were followed. The DNA digestion pattern showed a clear smearing at 20 min and 10 min for the “free MNase” and the CauNsi1-MNase strains, respectively (Fig. S2).

10.1128/mSphere.00646-20.2 FIG S2 Evaluation of genomic DNA fragmentation by agarose gel electrophoresis at 0, 0.5, 1, 2.5, 5, 10, 20, 60, and 120 min of calcium exposure in the CauNsi1^MNase^ and the free MNase strains in C. auris. L, 100-bp DNA ladder. Download FIG S2, TIF file, 0.3 MB. Copyright © 2020 Tebbji et al. 2020 Tebbji et al. This content is distributed under the terms of the Creative Commons Attribution 4.0 International license.

### Genome-wide binding of Nsi1 and Snf2 by ChEC-seq.

**FIG 2:**
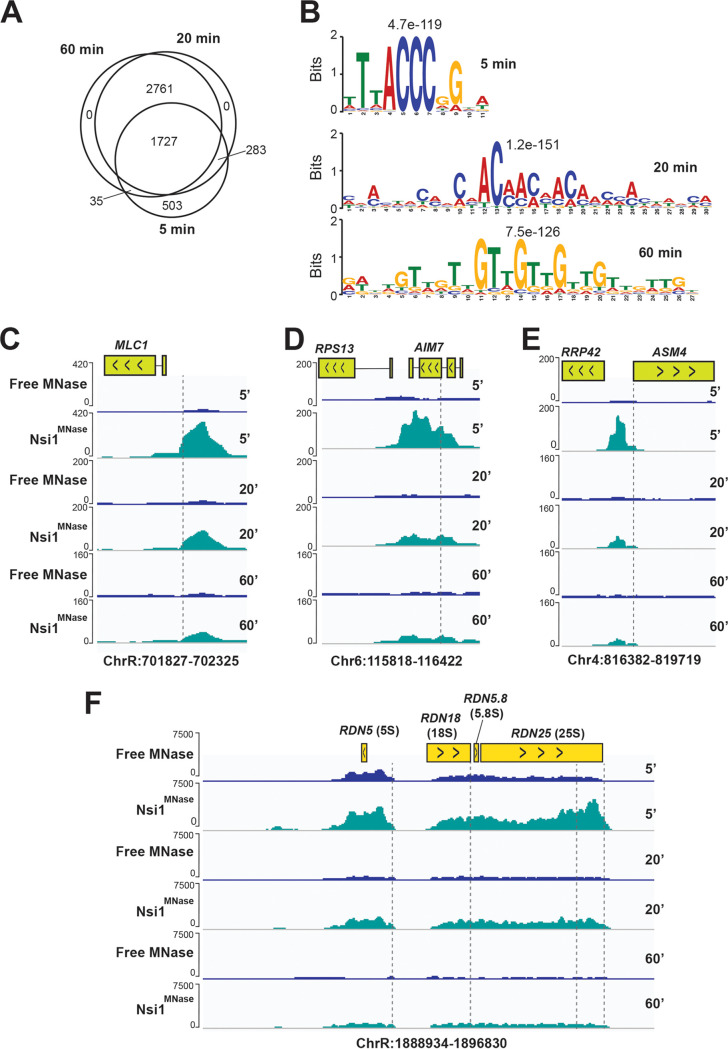
Genome-wide occupancy of the transcription factor Nsi1 with ChEC-seq. (A) Temporal analysis of Nsi1 binding events. Venn diagram showing the overlap of Nsi1 binding events at three distinct MNase activation times (5, 20, and 60 min). (B) Motif scores for Nsi1-bound promoters at 5, 20, and 60 min after MNase activation. The motif logos were generated using MEME-ChIP software on the 1,000 high-scoring peaks. (C to F) Snapshots of genomic regions showing the ChEC-seq signal for Nsi1^MNase^ and the free MNase strains at 5, 20, and 60 min after MNase activation. The positions of the Nsi1 motifs are indicated by the dashed lines. Nsi1 occupies the promoter of *MLC1* (C), *AIM7* (D), and *ASM4* (E), in addition to many sites within the rDNA locus (F).

To provide a proof of principle for using ChEC in *Candida* spp., we focused our effort on C. albicans. To assess the ChEC-seq performance in C. albicans, we have chosen to map the genomic occupancy of Nsi1, which is an ortholog of the DNA-binding protein Reb1 for which the genome-wide occupancy was previously established by ChEC-seq in S. cerevisiae (20). We detected 2,548, 4,771, and 4,523 Nsi1 peaks upon 5, 20, and 60 min MNase activation, respectively (see Table S2 in the supplemental material and Fig. 2A). *De novo* motif analysis of intergenic bound Nsi1 regions showed a significant enrichment of the bona fide Reb1/Nsi1 site (TTACCCGG) at 5 min, while a nonspecific long AC/TG-rich sequence was the most enriched at 20 and 60 min (Fig. 2B). This suggests that the 5 min MNase cleavage mapped the Nsi1 fast class binding events, while 20 and 60 min captured the slow class binding that lacks the robust consensus motif. Thus, as for S. cerevisiae, our ChEC-seq data recapitulated the time-dependent binding behavior of transcriptional regulators and can be used to map early high-affinity interactions with consensus motifs and sequence that are preferentially sampled by a given protein (20). While the fast high-scoring sites are robust binding events, the slow low-scoring sites should be interpreted with caution since they might recapitulate nonspecific MNase cleavages that are near high-scoring sites in accessible chromatin (37, 38). For instance, and to identify high-confidence slow sites, slow ChEC-seq binding events should be matched with the set of transcripts with altered levels in a TR mutant to assess whether TR occupancy correlates with gene expression alterations at bound loci. Our genome-wide occupancy data recapitulated the overall functions of either Nsi1 or Reb1 in S. cerevisiae as reflected by Nsi1 binding to the promoter of ribosome biogenesis and rRNA genes (Fig. 2C to F) (39–41).

**FIG 3:**
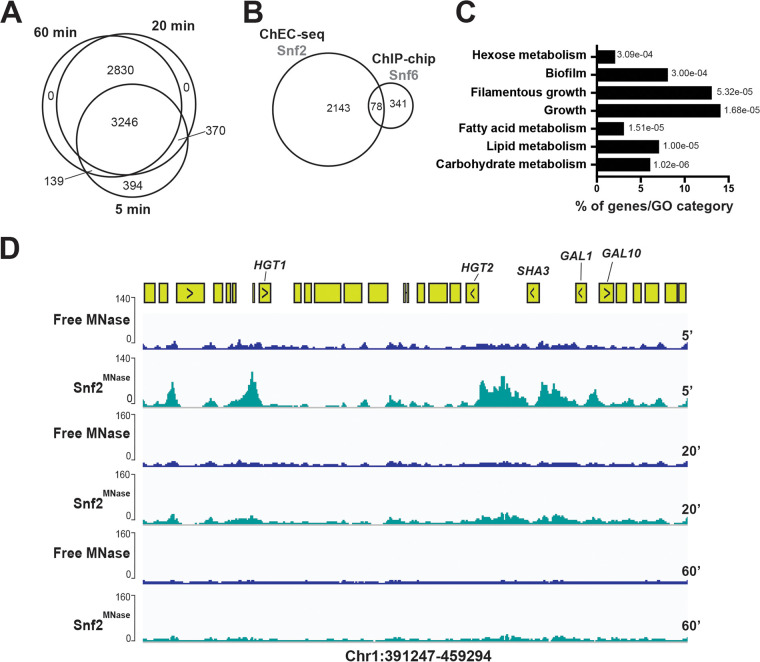
Genome-wide occupancy of the chromatin remodeler Snf2 with ChEC-seq. (A) Venn diagram showing the overlap of Snf2 promoter binding at 5, 20, and 60 min after MNase activation. (B) Comparison of SWI/SNF genomic occupancies by ChEC-seq and ChIP-chip method. Venn diagram of overlap between Snf2 ChEC-seq sites and Snf6 ChIP-chip using high-density tiling arrays. (C) Gene ontology of biological process associated with Snf2-bound promoters at 5 min after MNase activation. The *P* values were calculated using hypergeometric distribution as described in the GO Term Finder Tool website (http://candidagenome.org/cgi-bin/GO/goTermFinder). (D) Snapshot of genomic regions showing the ChEC-seq signal for Snf2^MNase^ and the free MNase strains at 5, 20, and 60 min after MNase activation. Genome browser view of Snf2 ChEC-seq displaying promoter occupancies of carbohydrate metabolism genes, including galactolysis (*GAL1* and *GAL10*) and hexose transport (*SHA3*, *HGT2*, and *HGT1*).

ChEC-seq of Snf2 identified 4,145, 6,446, and 6,215 peaks at 5, 20, and 60 min MNase cleavage, respectively, which is 10-fold higher than the number of peaks detected under similar growth conditions by ChIP-tiling array of the SWI/SNF subunit, Snf6 (7) (Fig. 3A and B and Table S3). As for Nsi1, the 20 and 60 min ChEC-seq data were similar and might capture the slow sites. The Snf2 fast bound promoters were enriched mainly in carbohydrate metabolism mirroring the previously characterized role of the SWI/SNF complex in C. albicans (Fig. 3C) (6, 7). Snf2 occupied promoters of hexose transport and carbon utilization genes (galactolysis) that were previously shown to be modulated by the SWI/SNF subunit Snf5 (6) (Fig. 3D).

### Conclusion.

We have constructed a new set of PCR-based MNase-tagging plasmids to map genomic occupancy of different transcriptional regulators in the human-pathogenic yeast C. albicans and other non-*albicans Candida* species. Compared to the other ChIP-based techniques, the ChEC procedure relies on total DNA extraction instead of chromatin solubilization and does not require protein-DNA cross-linking or sonication, thus avoiding artifacts related to epitope masking or the hyper-ChIPable euchromatic phenomenon (42, 43). So far, ChEC has been exclusively used in the model yeast S. cerevisiae to map chromatin occupancy of general transcriptional regulators (44), chromatin remodelers (27, 30, 45), and histone modifiers (31, 32) in addition to transcription factors (46, 47). As many transcriptional regulators and chromatin remodelers are key virulence and drug resistance factors in C. albicans and other fungi (6, 13, 17, 48–50), ChEC-seq represents an attracting tool to unbiasedly decipher transcriptional regulatory networks of fungal fitness.

## MATERIALS AND METHODS

### Strains, media, and growth assays.

C. albicans was routinely maintained at 30°C on YPD (1% yeast extract, 2% peptone, and 2% dextrose with 50 mg/ml uridine). The C. albicans wild-type (WT) strain SN148 (*his1*/*his1 leu2*/*leu2 arg4*/*arg4 ura3*/*ura3*::*imm434 IRO1*/*iro1*::*imm434*) (51) used in this study derives from the SC5314 clinical strain. For C. auris, the clinical B8441 strain (52) was used for *SAT1*-MNase tagging. For spot dilution assays, overnight cultures of both C. albicans and C. auris were diluted to an optical density at 600 nm (OD_600_) of 1 and 5-fold serial dilutions were prepared in distilled water. A total of 4 μl of each dilution was spotted on YPD agar plates for 1 day at different temperatures (30°C, 37°C, and 40°C) and imaged using the SP imager system. For growth assays in liquid media, overnight cultures of C. albicans and C. auris were resuspended in fresh YPD medium at an OD_600_ of 0.05 and added to a flat-bottom 96-well plate in a total volume of 100 μl per well in addition to the tested compounds (fluconazole [1 μg/ml] and sodium chloride [0.3 M]). Growth of the MNase-tagged and the control strains was also assessed in the synthetic complete (SC) medium. For each experiment, a compound-free and a cell-free negative control were included. Growth assay curves were performed in triplicate in 96-well plates using a Sunrise plate reader (Tecan) at 30°C with constant agitation with OD_600_ readings taken at 24 h. The relative growth was determined as the OD ratio of the YPD control to the treated cultures and is expressed as a percentage. The results are the means of the results from at least three biological replicates. Statistical significance was tested using Student’s *t* test.

### Construction of the pFA-MNase plasmids and the “free MNase” control strains.

The pFA-MNase-CaURA3, pFA-MNase-CaHIS1, and pFA-MNase-CaARG4 plasmids were constructed as follows. DNA of the 3xFLAG epitope-MNase module was synthesized by Biobasic, codon optimized for C. albicans (a total of 11 CTG codons of the MNase were changed to TTA or TTG). The PacI-AscI 3xFLAG-MNase fragment was cloned in the PacI-AscI-digested pFA-TAP-CaURA3, pFA-TAP-CaHIS1, and pFA-TAP-CaARG4 (33). For pFA-MNase-*SAT1*, pFA-MNase-Ca*URA3* was double digested with AscI and SacI restriction enzymes to remove the *URA3* auxotrophy marker. The *SAT1* marker was amplified from pFA-SAT1 (34) with primers (see Table S1 in the supplemental material) containing restriction sites AscI-SacI and cloned into the AscI-SacI-digested pFA-MNase. The resulting pFA-MNase-SAT1 was sequenced to confirm the integrity of the Sat1 dominant marker.

10.1128/mSphere.00646-20.3 TABLE S1 List of primers used in this study. Download Table S1, XLSX file, 0.02 MB. Copyright © 2020 Tebbji et al. 2020 Tebbji et al. This content is distributed under the terms of the Creative Commons Attribution 4.0 International license.

10.1128/mSphere.00646-20.4 TABLE S2 List of Nsi1 binding peaks. Download Table S2, XLSX file, 0.3 MB. Copyright © 2020 Tebbji et al. 2020 Tebbji et al. This content is distributed under the terms of the Creative Commons Attribution 4.0 International license.

10.1128/mSphere.00646-20.5 TABLE S3 List of Snf2 binding peaks. Download Table S3, XLSX file, 0.4 MB. Copyright © 2020 Tebbji et al. 2020 Tebbji et al. This content is distributed under the terms of the Creative Commons Attribution 4.0 International license.

The free MNase control strain was constructed as follows. The C. albicans codon-optimized DNA of the 3xFLAG-MNase-nuclear localization signal (SV40) (3xFLAG-MNase-SV40) construct delimited by NheI and MluI restriction sites was synthesized. The NheI-MluI-digested 3xFLAG-MNase-SV40 fragment was then cloned into the CIp-p*ACT1*-CYC vector (53) to ensure constitutive expression of MNase in C. albicans. The CIp-p*ACT1*-3xFLAG-MNase-SV40-CYC plasmid was linearized by StuI restriction enzyme and integrated at the *RPS1* locus of the SN148 WT strain.

For the C. auris MNase control strain, the *URA3* auxotrophy marker of the CIp-p*ACT1*-3xFLAG-MNase-SV40-CYC was replaced by SAT1 as follows. The *SAT1* marker was amplified from pFA-SAT1 with primers containing restriction sites NotI-NheI (Table S1) and cloned into the NotI-NheI-digested CIp-p*ACT1*-3xFLAG-MNase-SV40-CYC. To allow the integration of the CIp-p*ACT1*-3xFLAG-MNase-SV40-CYC in the C. auris genome, the C. albicans *RPS1* integrative locus was replaced by a short 900-bp C. auris intergenic region *CauNI* (C. auris neutral intergenic; GenBank accession no. PEKT02000001: 871,442 to 872,342) as follows. The *CauNI* region was amplified from the C. auris B8441 genomic DNA with primers containing restriction sites SacII-NotI (Table S1) and cloned into the SacII*-*NotI-digested CIp-p*ACT1*-3xFLAG-MNase-SV40-CYC-*SAT1* plasmid. The resulting plasmid was linearized by SacII restriction enzyme and integrated at the *CauNI* locus of the C. auris B8441 strain. The correct integration of the CIp-p*ACT1*-3xFLAG-MNase-SV40-CYC-*SAT1* cassette was verified by PCR. Integration of any exogenous DNA or the CIp-p*ACT1*-3xFLAG-MNase-SV40-CYC-*SAT1* at the *CauNI* has no impact on the *in vitro* growth of C. auris.

### PCR-based tagging of endogenous loci in C. albicans and C. auris.

*SNF2* (C2_02100W_A) and *NSI1* (C6_03550C_A) were MNase tagged *in vivo* with the MNase cassette PCR products following the protocol described by Lavoie et al. (33). The MNase cassettes were amplified using a 120-bp primer pair with 20 bp of vector sequences (forward [GGTCGACGGATCCCCGGGTT] and reverse [TCGATGAATTCGAGCTCGTT]) and 100 bp from *SNF2* (C2_02100W_A) and *NSI1* (C6_03550C_A) (Table S1). PCRs were performed in 50-μl volumes with 1 ng pFA-MNase plasmid and the Q5 high-fidelity polymerase (New England Biolabs). PCR thermocycling was executed as follows: (i) initial denaturing, 98°C for 30 s; (ii) 10 cycles with 1 cycle consisting of 98°C for 10 s, 50°C for 1 min, and 72°C for 3 min; and (iii) 25 cycles with 1 cycle consisting of 98°C for 30 s, 55°C for 1 min, and 72°C for 3 min. PCR products were used directly to transform the WT strain SN148 using a lithium acetate transformation protocol (54). Transformants were selected on selective plates, and positive colonies were analyzed by PCR to confirm the correct integration of the MNase tag. For C. auris, *CauNSI1* (B9J08_003000) was MNase tagged *in vivo* with the MNase-*SAT1* cassette as described for C. albicans with the exception that a reverse vector sequence of 24 bp was used (TCTGATATCATCGATGAATTCGAG).

### ChEC-seq procedure.

For each ChEC experiment, saturated overnight cultures of C. albicans MNase tagged and free MNase strains were diluted to a starting OD_600_ of 0.1 in 50 ml YPD medium and grown at 30°C to an OD_600_ of 0.7 to 0.8. Cells were pelleted at 3,000 × *g* for 5 min and washed three times with 1 ml buffer A (15 mM Tris [pH 7.5], 80 mM KCl, 0.1 mM EGTA, 0.2 mM spermine, 0.5 mM spermidine, one tablet Roche cOmplete EDTA-free mini protease inhibitors, 1 mM phenylmethylsulfonyl fluoride [PMSF]). Cells were then resuspended in 800 μl buffer A containing 0.1% digitonin (Sigma) and permeabilized for 10 min at 30°C with shaking. MNase digestions were performed by adding CaCl_2_ to a final concentration of 5 mM and incubated for the indicated time at 30°C. At each time point, a total of 200-μl aliquots of the ChEC digestions were transferred to a tube containing 50 μl of 250 mM EGTA to quench MNase digestion. For each factor analyzed, the time point zero corresponds to a condition where MNase was not activated by CaCl_2_. Nucleic acids were extracted using MasterPure yeast DNA purification kit (Epicentre, MPY80200) according to the manufacturer’s instructions and resuspended in 50 μl of 10 mM Tris-HCl buffer, pH 8.0. RNAs were digested with 10 μg RNase A at 37°C for 20 min. To assess MNase activity, 5 μl of digested DNA of each ChEC time point (time after CaCl_2_ addition) was loaded on a 1.5% agarose gel. ChEC DNA was subjected to size selection using the Pippin Prep (SageScience) size selection system with a 2% agarose gel cassette, allowing the removal of multikilobase genomic DNA fragments and the enrichment of 100- to 400-bp DNA fragments. For C. auris, the ChEC procedure was the same as for C. albicans except that MNase activation was performed at 37°C.

### Library preparation, next-generation sequencing (NGS), and peak calling.

The NEBNext Ultra II DNA Library Prep kit for Illumina was used to construct the ChEC-seq library following the manufacturer’s instruction. The quality, quantity, and size distribution of the libraries were determined using an Agilent bioanalyzer. A 50-bp single-end sequencing of DNAs was performed using an Illumina HiSeq 4000 sequencing system. Sequences were trimmed to remove adapters using TRIMMOMATIC with options “TRAILING:30” (55). Reads thus obtained were mapped to the C. albicans genome (*Candida*_*albicans*_SC5314 assembly 22) (56) using Bowtie2 with “-q --phred33 --no-unal” options (57). Peaks were determined using MACS2 algorithm (58) with options “-BAM -nomodel-extsize 200-keep-dup all.” MACS2 outputs BED6+4 format files that contain the peak locations (narrowPeak) and peak summit locations for each peak. The biological replicates were merged into single samples, retaining all high-confidence peaks (q-value cutoff = 0.05) from all replicates. Read alignment, peaks, and track visualization using bedgraph files were performed as previously described (20, 44). BigWig files were also generated to visualize the different ChEC-seq tracks on the Integrative Genomics Viewer (IGV) (https://igv.org) interface. *cis*-Regulatory motif enrichment was assessed in the top high-scoring 1,000 peaks for both Nsi1 and Snf2 using MEME-ChIP software (59).

### Data availability.

The sequences of plasmids pFA-MNase-CaHIS1, pFA-MNase-CaARG4, pFA-MNase-CaURA3, and pFA-MNase-SAT1 have been submitted to GenBank and have been assigned the following accession numbers: MT181237, MT181238, MT181239, and MT223485. All ChEC-seq data generated in this study were submitted to GEO database under the accession number GSE150063.
