# Supplementary figures and images for: High-Resolution Genome-Wide Occupancy in Candida spp. Using ChEC-seq
Source: mSphere. 2020 Oct 14;5(5):e00646-20. doi: 10.1128/mSphere.00646-20 (PMC7565893; doi:10.1128/mSphere.00646-20)

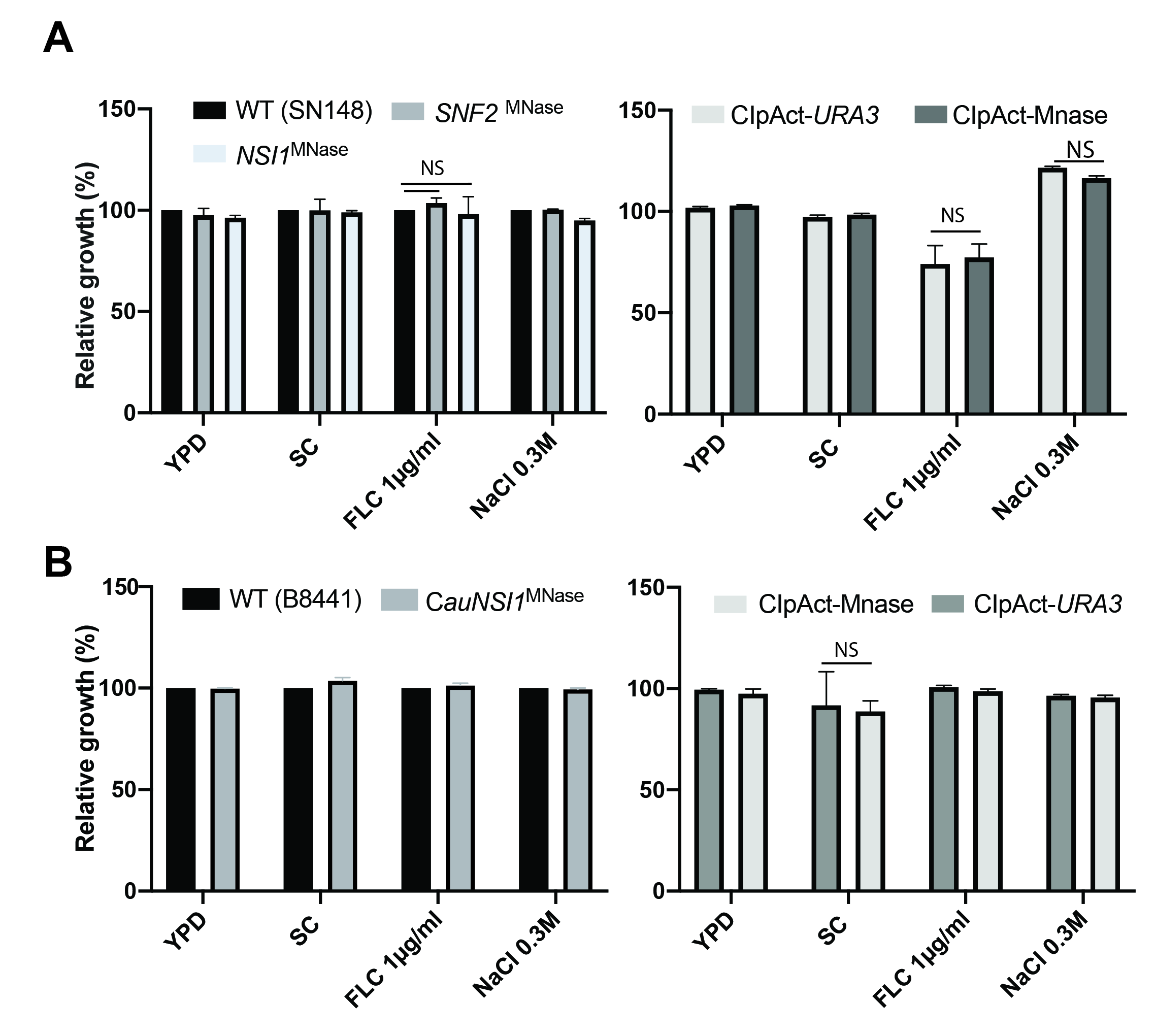

Supplement: FIG S1 [file mSphere.00646-20-sf001.tif]

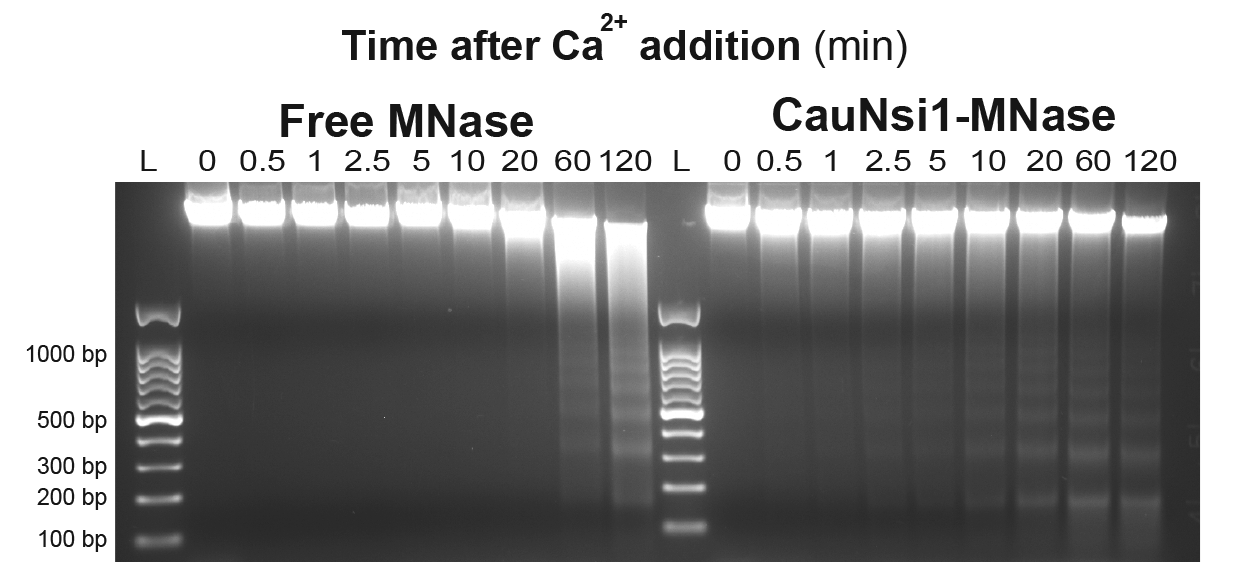

Supplement: FIG S2 [file mSphere.00646-20-sf002.tif]
